# Supplementary material for: A maize epimerase modulates cell wall synthesis and glycosylation during stomatal morphogenesis
Source: Nat Commun. 2023 Jul 20;14:4384. doi: 10.1038/s41467-023-40013-6 (PMC10359280; doi:10.1038/s41467-023-40013-6)
Supplement: Supplementary file 1 — Supplementary Information [file 41467_2023_40013_MOESM1_ESM.pdf]

Supplementary information

Title: A maize epimerase modulates cell wall synthesis and glycosylation during stomatal morphogenesis

Authors: Yusen Zhou[1,2,8], Tian Zhang[1,8], Xiaocui Wang[3,4,8], Wenqiang Wu[1,8], Jingjing Xing[1], Zuliang Li[1], Xin Qiao[1], Chunrui Zhang[3,4], Xiaohang Wang[1], Guangshun Wang[1], Wenhui Li[2], Shenglong Bai[1], Zhi Li[1], Yuanzhen Suo[5], Jiajia Wang[6], Yanli Niu[1], Junli Zhang[1], Chen Lan[1], Zhubing Hu[1,2], Baozhu Li[1], Xuebin Zhang[1], Wei Wang[1], David W. Galbraith[1,7], Yuhang Chen[3,4], Siyi Guo[1,2,*] and Chun-Peng Song[1,2,*].

*To whom correspondence should be addressed. Email: songcp@henu.edu.cn; guosiyi@henu.edu.cn

Supplementary Figure S1

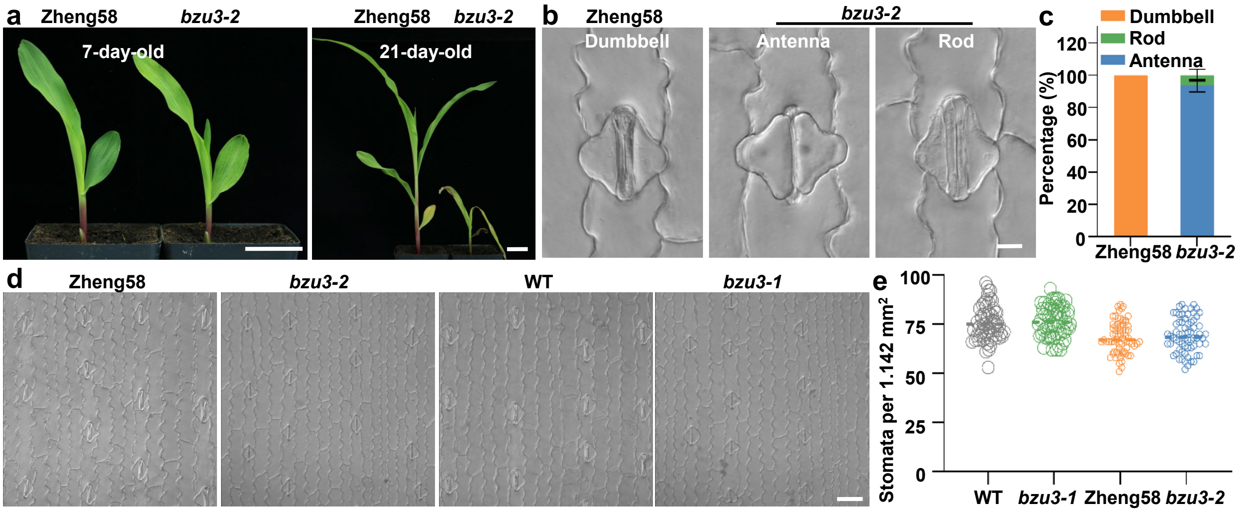

Fig. S1 The guard cell phenotype of the *bzu3-2* mutant is similar to that of *bzu3-1*.

a Representative images of 7- and 21-day-old seedlings of Zheng58 and *bzu3-2*. Scale bars, 3 cm.

b Representative differential interference contrast (DIC) images of stomata in Zheng58, *bzu3-2* (the second leaf of 7-day-old seedlings). Scale bar, 10 μm.

c Proportional phenotypic scoring analysis of stomatal types in Zheng58 and *bzu3-2.* The data was obtained by counting guard cells from ten seedlings of each genotype (Zheng58: *n* = 1108, *bzu3-2*: *n* = 1286). Values represent means ± SD. SD, standard deviation.

d Epidermis from Zheng58, *bzu3-2,* Wild type (WT), and *bzu3-1,* as observed by a DIC microscope at lower magnification. Scale bar, 50 μm.

e Stomatal density analysis of Zheng58, *bzu3-2,* WT, and *bzu3-1.* The area of the vision field was 1.142 mm^2. Data represent means ± SD. SD, standard deviation. (*n* = 10 individuals per genotype, the second leaf of 7-day-old seedlings, taking 5-7 pictures of each leaf.)

Source data are provided as a Source Data file.

Supplementary Figure S2

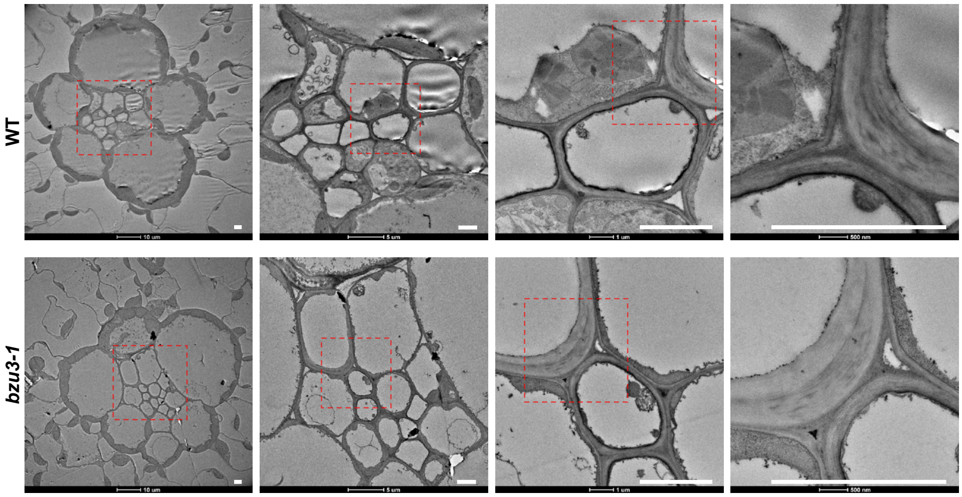

Fig. S2 Wall thickening of leaf vascular bundles is similar between the WT and *bzu3-1* mutant.

Leaf vascular bundles were observed in 7-day-old seedlings via transmission electron microscopy**.** The square dashed boxes (red) indicate enlarged regions of the three panels from left to right, respectively. Scale bars, 3 μm.

Supplementary Figure S3

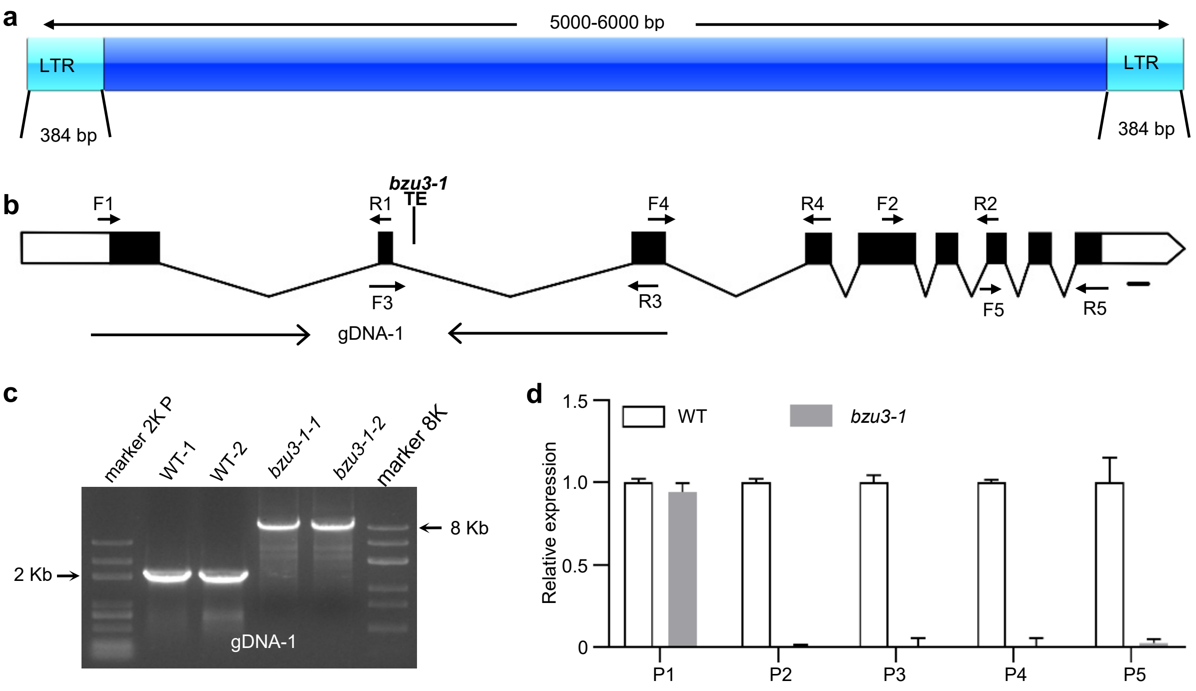

Fig. S3 Molecular identification of *bzu3-1* mutants.

a A schematic diagram of the site of transposon insertion in the *bzu3-1* mutant. The viral retrotransposon contains a full-length 5-6 kb sequence containing forward long terminal repeats (LTR). The LTR length is 384 bp.

b Schematic diagram of the *BZU3* genomic structure with the positions of the RT-qPCR primers (P1 (F1 and R1), P2 (F2 and R2), P3 (F3 and R3), P4 (F4 and R4), P5 (F5 and R5)) and identification primers (gDNA-1 (F1 and R3)) of the *bzu3-1* mutant.

c Molecular verification of *bzu3-1* mutants. Genomic PCR using primers (F1 and R3) was conducted using two independent plants of WT and *bzu3-1* mutants, the PCR products being separated on a 1% agarose gel. Experiments were repeated three times with similar results.

d Transcript enrichment of *BZU3* was detected by RT-qPCR. RT-qPCR was conducted using 7-day-old seedlings and the primers indicated in (**b**). *ZmUbiquitin* was used as an internal control. Values are means ± SD (n = 3). SD, standard deviation.

Source data are provided as a Source Data file.

Supplementary Figure S4

a

```
BZU3          MVSAVLRTILVTGGAGYIGSHTVLQLLQQGFRVVVVDNLDNASEAALARVAELAGHDGANLVFHKVDLRDRHALVDIFSSHRF   83
BZU3bzu3-2    MVSAVLRTILVTGGAGYIGSHTVLQLLQQGFRVVVVDNLDNASEAALARVAELAGHDGANLVFHKVDLRDRHALVDIFSSHRF   83
consensus     mvsavlrtilvtggagyigshtvlqllqqgfrvvvvdnldnaseaalarvaelaghdganlvfhkvdlrdrhalvdifsshrf

BZU3          EAVIHFAGLKAVGESVHKPLLYYDNNLVGTITLLEVMAANGCKKLVFSSSATVYGWPKEVPCTEEFPLCATNPYGRTKLVIED  166
BZU3bzu3-2    EAVIHFAGLKAVGESVHKPLLYYDNNLVGTITLLEVMAANGCKKLVFSSSATVYGWPKEVPCTEEFPLCATNPYGRTKLVIED  166
consensus     eavihfaglkavgesvhkpllyydnnlvgtitllevmaangckklvfsssatvygwpkevpcteefplcatnpygrtklvied

BZU3          ICRDVHRSDPDWKIILLRYFNPVGAHPSGYIGEDPCGVPNNLMPYVQQVAVGRLPHLTVYGTDYSTKDGTGVRDYIHVVDLAD  249
BZU3bzu3-2    ICRDVHRSDPDWKIILLRYFNPVGAHPSGYIGEDPCGVPNNLMPYVQQVAVGRLPHLTVYGTDYSTKDGTGVRDYIHVVDLAD  249
consensus     icrdvhrsdpdwkiillryfnpvgahpsgyigedpcgvpnnlmpyvqqvavgrlphltvygtdystkdgtgvrdyihvvdlad

BZU3          GHIAALRKLHEDSDKIGCEVYNLGTGKGTSVLEMVAAFEKASGKKIPLVFAGRRPGDAEIVYAATAKAEKELKWKAKYG...I  329
BZU3bzu3-2    GHIAALRKLHEDSDKIGCEVYNLGTGKGTRCWKWWLHSRRLLGRKSLWCSLGEDPETQRSSTPQLPRQRKSSNGRPSTGSRRC  332
consensus     ghiaalrklhedsdkigcevynlgtgkgt             g k      g  p              k        g

BZU3          EEMCRDLWNWASKNPYGYAGSRDNSK.......................  355
BZU3bzu3-2    AEICGTGRARTRTATLGHATTANEPPIHASHPAVGASSSLIAYATIIN  380
consensus      e c            g a
```

b

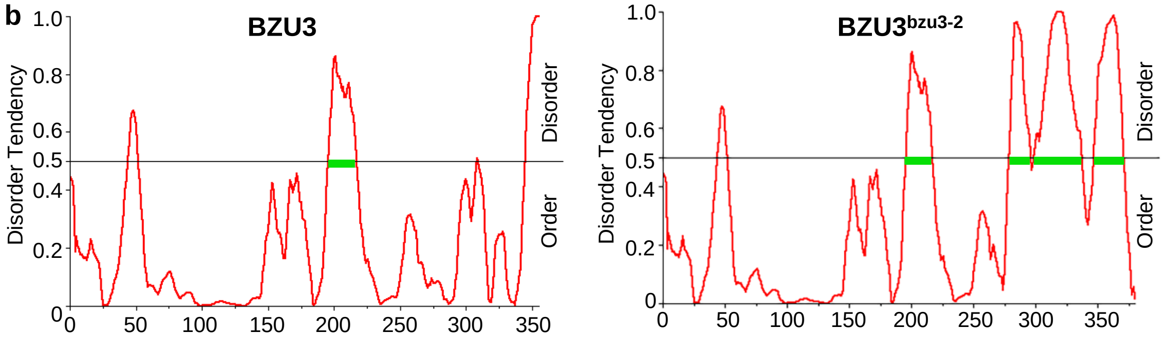

Fig. S4 Characteristics and alignment of BZU3 and BZU3$^{bzu3\text{-}2}$ (the BZU3 mutant protein from *bzu3-2*).

a Amino acid alignment analysis of the BZU3 and BZU3$^{bzu3\text{-}2}$ proteins.

b BZU3$^{bzu3\text{-}2}$ showed more disorder than BZU3. We predicted intrinsic disorder tendency using the PONDR tutorial (http://www.pondr.com/) applied to the entire length of BZU3 (left panel) and BZU3$^{bzu3\text{-}2}$ (right panel). Detailed methods can be found in the PONDR tutorial. The most disordered regions of the BZU3 (residues 196-217) and BZU3$^{bzu3\text{-}2}$ (residues 196-217, residues 279-296, residues 299-337, and residues 347-371) were indicated with bold green lines.

Supplementary Figure S5

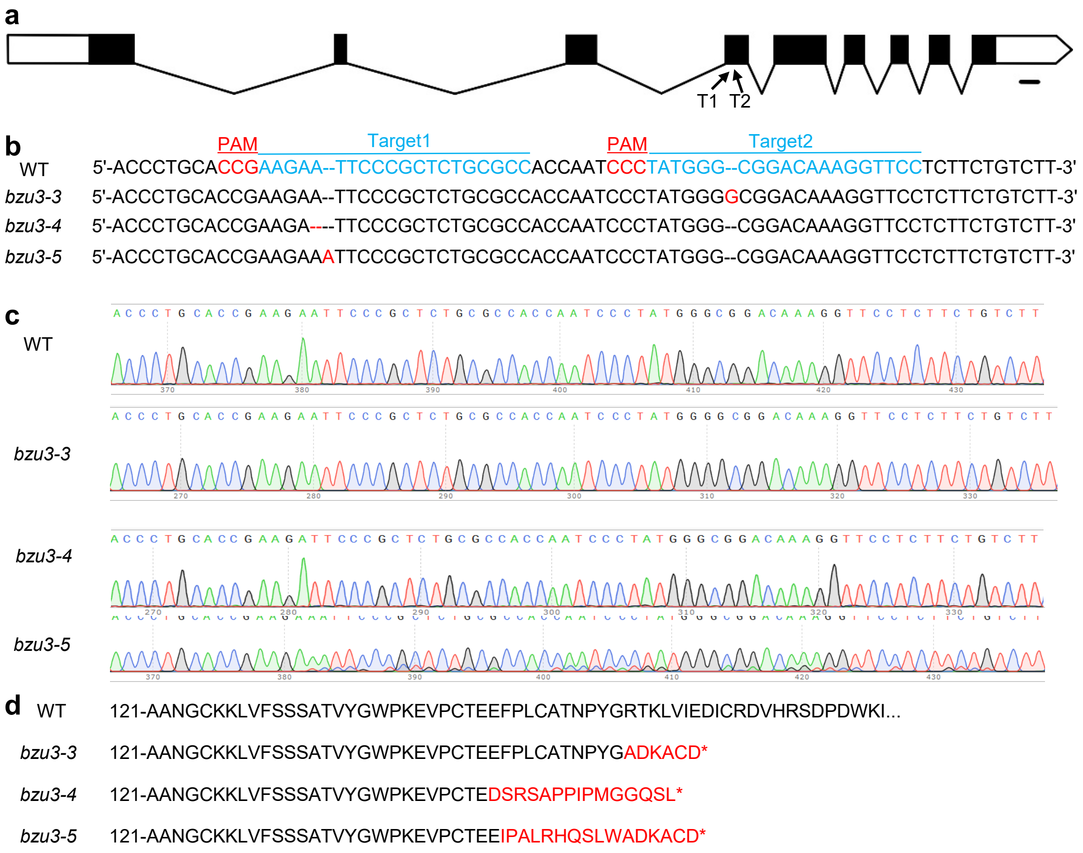

Fig. S5 The CRISPR/Cas9 alleles *bzu3-3*, *bzu3-4*, and *bzu3-5*.

a Schematic representation of *BZU3* genomic structure. The black arrowheads indicate two sgRNA target sites (T1 and T2).

b The sequences of WT and CRISPR-Cas9-induced (*bzu3-3*, *bzu3-4*, and *bzu3-5*) mutations.

c Sequence chromatograms of WT and CRISPR-Cas9-induced (*bzu3-3*, *bzu3-4*, and *bzu3-5*) mutations.

d Amino acids corresponding to the WT and CRISPR-Cas9-induced (*bzu3-3*, *bzu3-4*, and *bzu3-5*) mutations.

Supplementary Figure S6

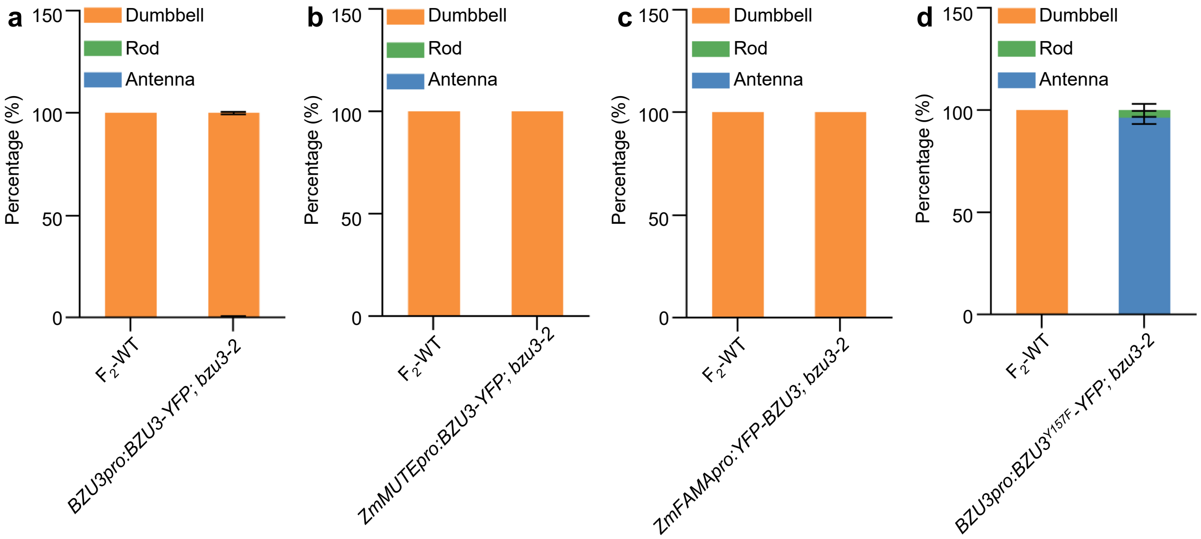

Fig. S6 Proportional phenotypic scoring analysis of complementation of the *bzu3-2* stomatal phenotype in transgenic plants expressing different promoter-driven *BZU3* constructions. (a) *BZU3pro:BZU3-YFP*, (b) *ZmMUTEpro:BZU3-YFP,* (c) ***ZmFAMApro:YFP-BZU3,* and** (d) ***BZU3pro:BZU3^{Y157F}-YFP.*** The data was obtained by counting guard cells from ten seedlings of each genotype (*n* = 1056, 1209, 1429, 1282, 1238, 1348, 1083, and 1158 respectively). Values represent means ± SD. SD, standard deviation. Source data are provided as a Source Data file.

Supplementary Figure S7

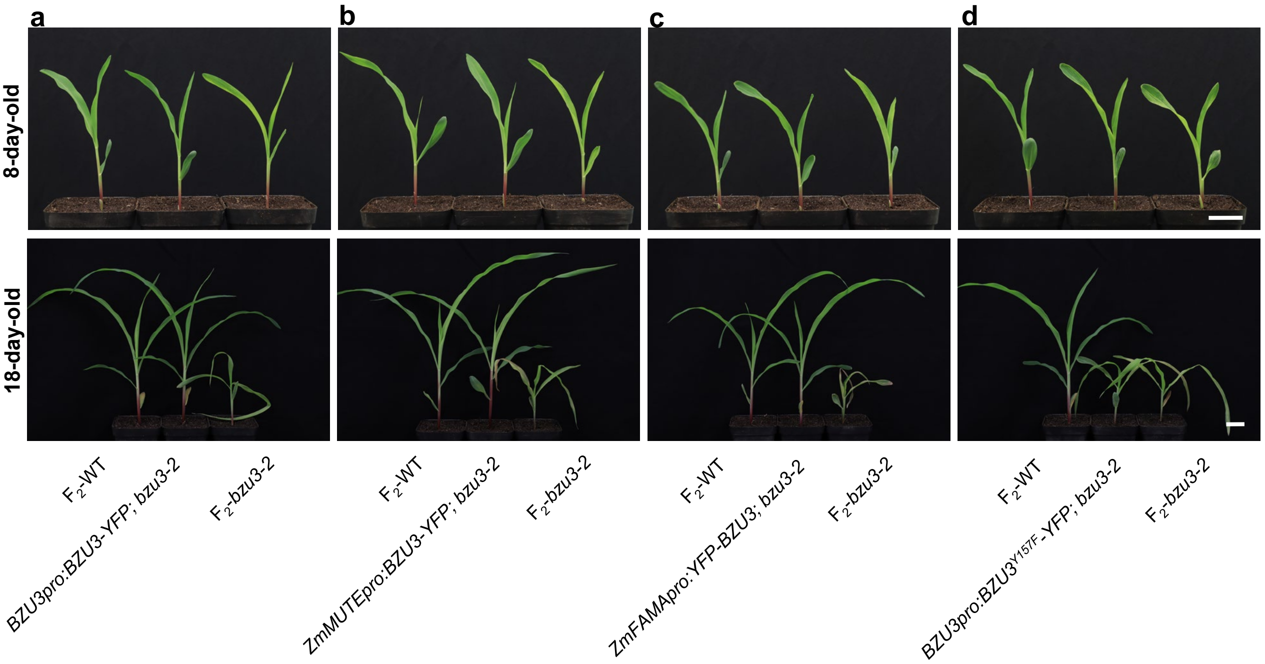

Fig. S7 Representative images of 8- and 18-day-old seedlings of WT, *bzu3-2*, *BZU3pro:BZU3-YFP; bzu3-2*, *ZmMUTEpro:BZU3-YFP; bzu3-2*, *ZmFAMApro:YFP-BZU3; bzu3-2*, and *BZU3pro:BZU3^{Y157F}-YFP; bzu3-2*.

BZU3pro:BZU3-YFP; bzu3-2 (**a**), *ZmMUTEpro:BZU3-YFP; bzu3-2* (**b**), *ZmFAMApro:YFP-BZU3; bzu3-2* (**c**), and *BZU3pro:BZU3^{Y157F}-YFP; bzu3-2* (**d**) represented *BZU3pro:BZU3-YFP*, *ZmMUTEpro:BZU3-YFP*, *ZmFAMApro:YFP-BZU3*, and *BZU3pro:BZU3^{Y157F}-YFP* complementing *bzu3-2* lines, respectively. Scale bars, 3 cm.

Supplementary Figure S8

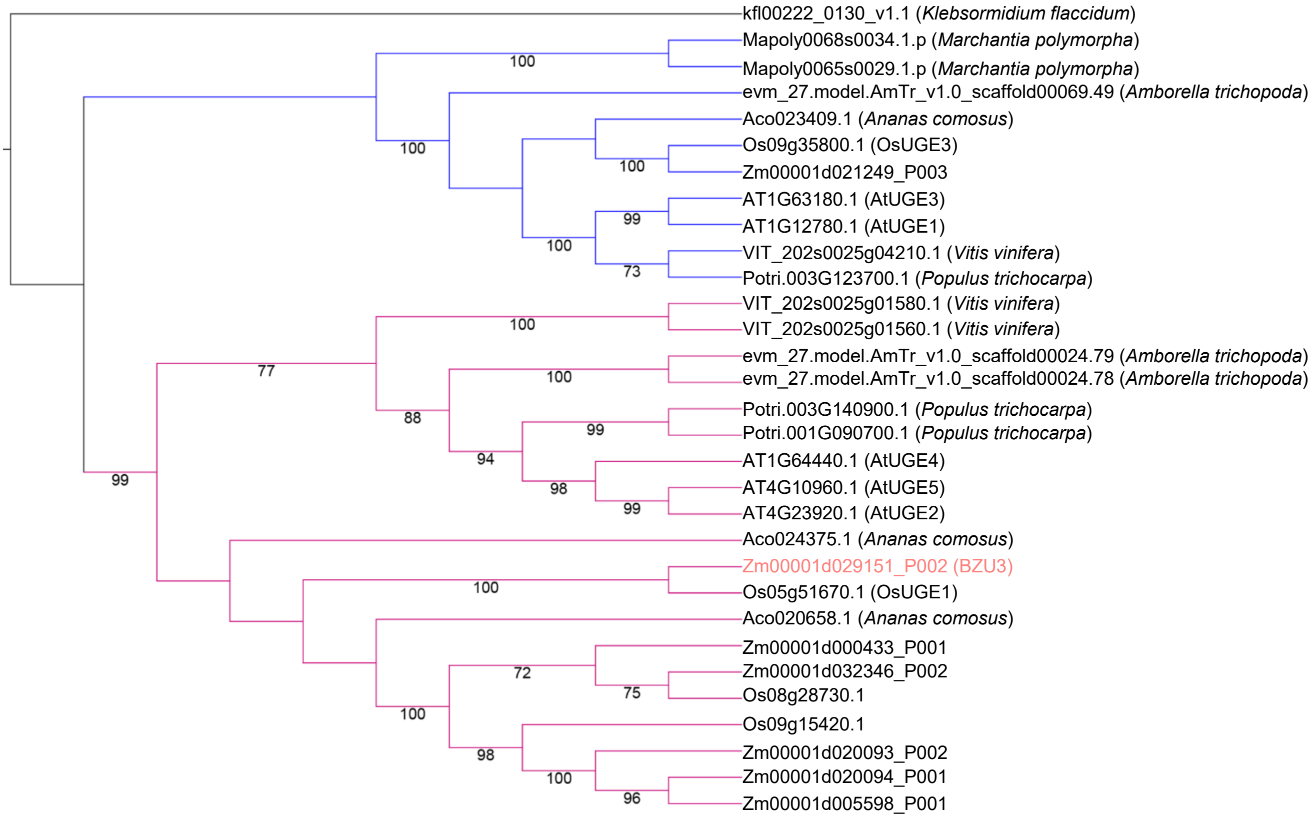

Fig. S8 Phylogenetic tree of UGEs in maize and other eukaryotes.

Phylogenetic analyses were performed by Iqtree, with 1,000 bootstraps using the ultrafast bootstrap approximation method. Numbers associated with branches indicated bootstrap percentages from maximum-likelihood analysis. Values for branches lower than 70% are not shown. Sequences used for phylogenetic analysis were downloaded from https://phytozome-next.jgi.doe.gov/ and http://www.plantmorphogenesis.bio.titech.ac.jp/~algae_genome_project/klebsormidium/.

Supplementary Figure S9

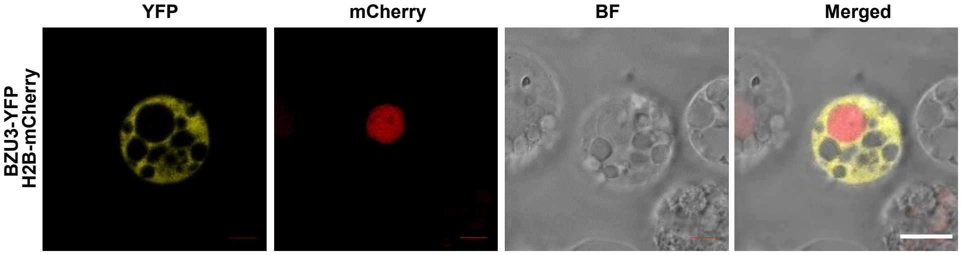

Fig. S9 Subcellular localization of BZU3.

Representative confocal microscopy images of BZU3-YFP fusion protein in maize mesophyll protoplasts are shown. *BZU3pro:BZU3-YFP* and *35S:H2B-mCherry* were transiently expressed in maize mesophyll protoplasts. Fluorescence was observed using a Zeiss confocal LSM710. Scale bar, 10 μm.

Supplementary Figure S10

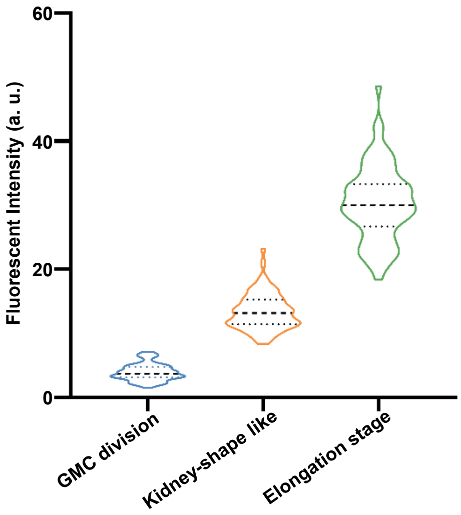

Fig. S10 BZU3 expression levels during stomatal development.

BZU3 expression levels were indicated by BZU3-YFP fluorescence intensity. BZU3-YFP signal was quantified at three stages: the GMC division stage (*n* = 79), the kidney-shape like stage (*n* = 67), and the elongation stage (*n* = 67). Images were taken using a Zeiss confocal LSM710 and fluorescence intensities were analyzed with ImageJ software (mean ± SD). SD, standard deviation. Source data are provided as a Source Data file.

Supplementary Figure S11

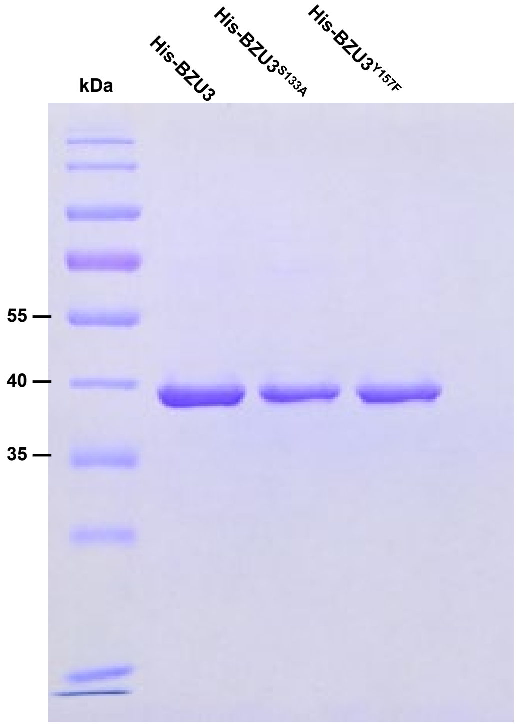

Fig. S11 Purification of BZU3 proteins.

His-BZU3, His-BZU3^{S133A} , and His-BZU3^{Y157F} fusion proteins were expressed and purified using Ni-NTA beads. The recombinant proteins were analyzed using SDS-PAGE, and stained with Coomassie brilliant blue G250. Source data are provided as a Source Data file.

Supplementary Figure S12

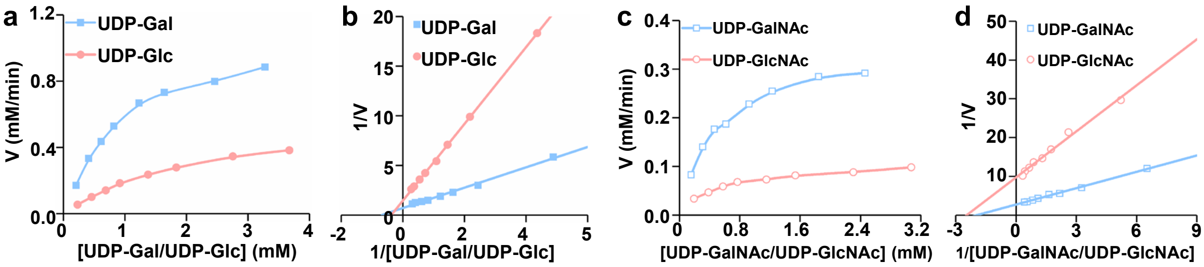

Fig. S12 Enzyme kinetics of BZU3 catalyzing conversions of UDP-Gal, UDP-Glc, UDP-GalNAc, and UDP-GlcNAc.

a Effect of the substrate concentrations of UDP-Gal or UDP-Glc on the enzyme activity of BZU3. Reactions were carried out with 25 nM of BZU3 in reaction buffer containing various concentrations of UDP-Gal or UDP-Glc at 30℃ for 1 min. Reaction products were quantified by HPLC as described in the methods.

b Lineweaver-Burk plots for UDP-Gal and UDP-Glc. The original data were from (**a**).

c Effect of the substrate concentrations of UDP-GalNAc or UDP-GlcNAc on the enzyme activity of BZU3. Reactions were carried out with 25 nM of BZU3 in reaction buffer containing various concentrations of UDP-GalNAc or UDP-GlcNAc at 30℃ for 1 min. Reaction products were quantified by HPLC as described in the methods.

d Lineweaver-Burk plots for UDP-GalNAc and UDP-GlcNAc. The original data were from (**c**).

Source data are provided as a Source Data file.

Supplementary Figure S13

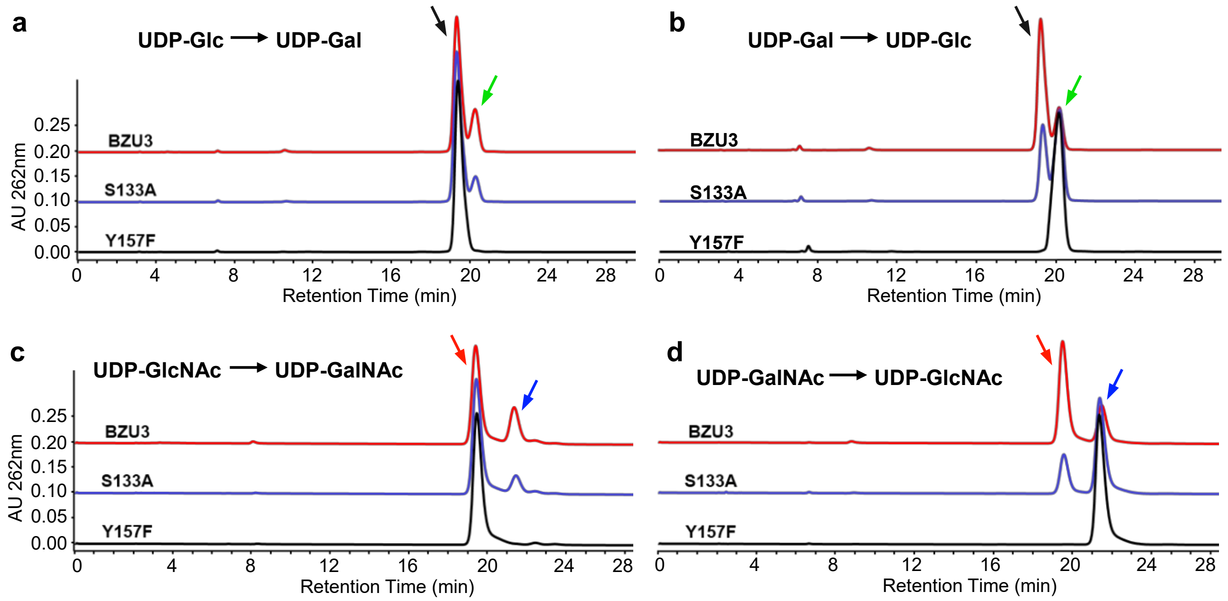

Fig. S13 Mutagenesis of key amino acids of BZU3 inactivated enzyme activity.

a HPLC detection of the enzyme activities of BZU3 and different BZU3 mutations (S133A, Y157F) as UDP-glucose 4-epimerases catalyzing the conversion of UDP-Glc to UDP-Gal at 30℃ for 30 min.

b HPLC detection of the enzyme activities of BZU3 and different BZU3 mutations (S133A, Y157F) as UDP-glucose 4-epimerases catalyzing conversion of UDP-Gal to UDP-Glc at 30℃ for 30 min. The retention times are 19.2 min for UDP-Glc and 20.2 min for UDP-Gal. The black and green arrowheads represent UDP-Glc and UDP-Gal, respectively.

c HPLC detection of the enzyme activities of BZU3 and different BZU3 mutations (S133A, Y157F) as UDP-glucose 4-epimerases catalyzing conversion of UDP-GlcNAc to UDP-GalNAc at 30℃ for 30 min.

d HPLC detection of the enzyme activities of BZU3 and different BZU3 mutations (S133A, Y157F) as UDP-glucose 4-epimerases catalyzing the conversion of UDP-GalNAc to UDP-GlcNAc at 30℃ for 30 min. The retention time is 20.6 min for UDP-GlcNAc and 22.2 min for UDP-GalNAc. The red and blue arrowheads represent UDP-GlcNAc and UDP-GalNAc, respectively.

Supplementary Figure S14

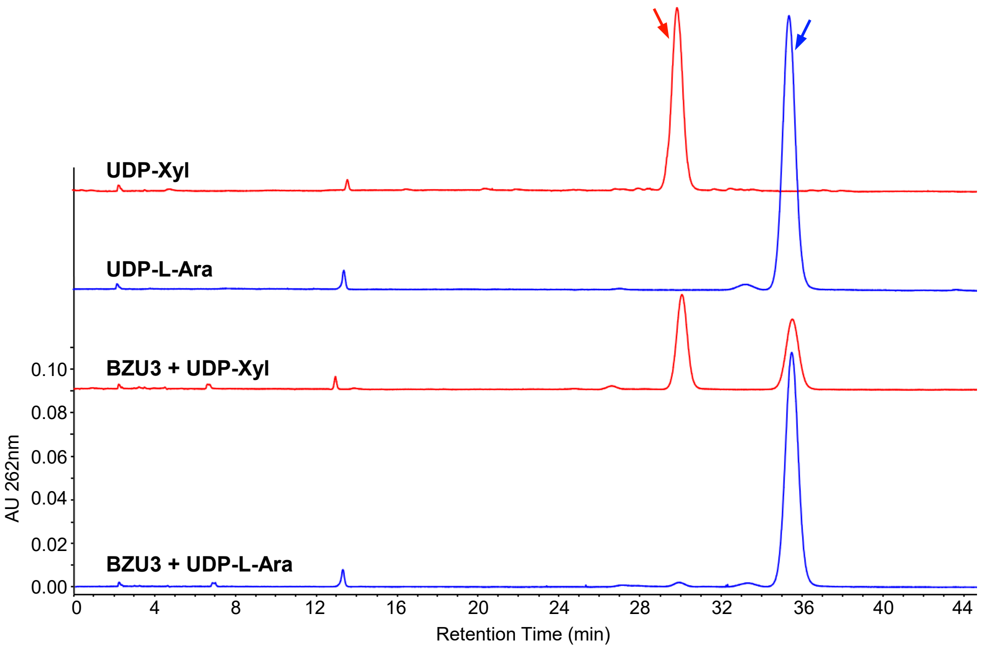

Fig. S14 HPLC detection of the enzyme activities of BZU3 catalyzing UDP-Xyl and UDP-L-Ara. BZU3 can convert about 45% of UDP-Xyl to UDP-L-Ara, but BZU3 is almost incapable of converting UDP-L-Ara to UDP-Xyl at 30℃. The retention time is 30.1 min for UDP-Xyl and 35.6 min for UDP-L-Ara. The red arrowheads represent UDP-Xyl. The blue arrowheads represent UDP-L-Ara. UDP-Xyl, UDP-Xylose. UDP-L-Ara, UDP-L-Arabinose.

Supplementary Figure S15

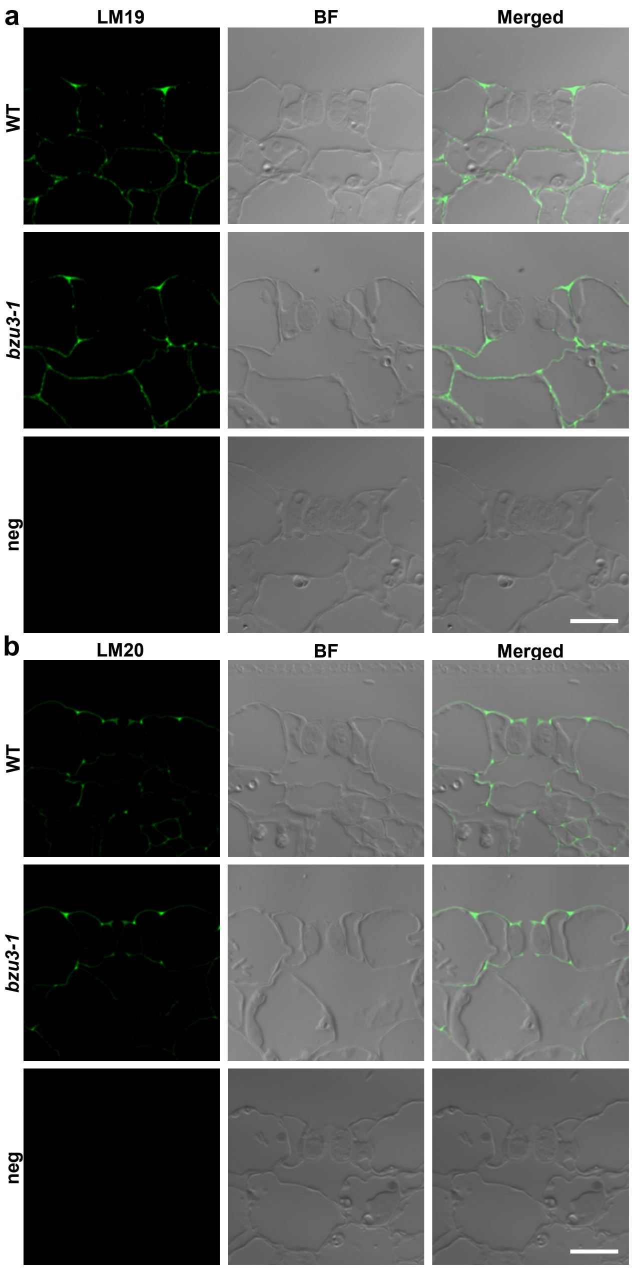

Fig. S15 Homogalacturonan (HGA) was barely present in GC walls.

a Representative images of HGA (no or little esterification) immunolabeling with LM19 antibody in resin-embedded sections (1.5 μm thick) of leaf base. neg, negative control. Scale bar, 10 μm.

b Representative images of HGA (highly methyl-esterified) immunolabeling with LM20 antibody in resin-embedded sections (1.5 μm thick) of leaf base. neg, negative control. Scale bar, 10 μm.

Supplementary Figure S16

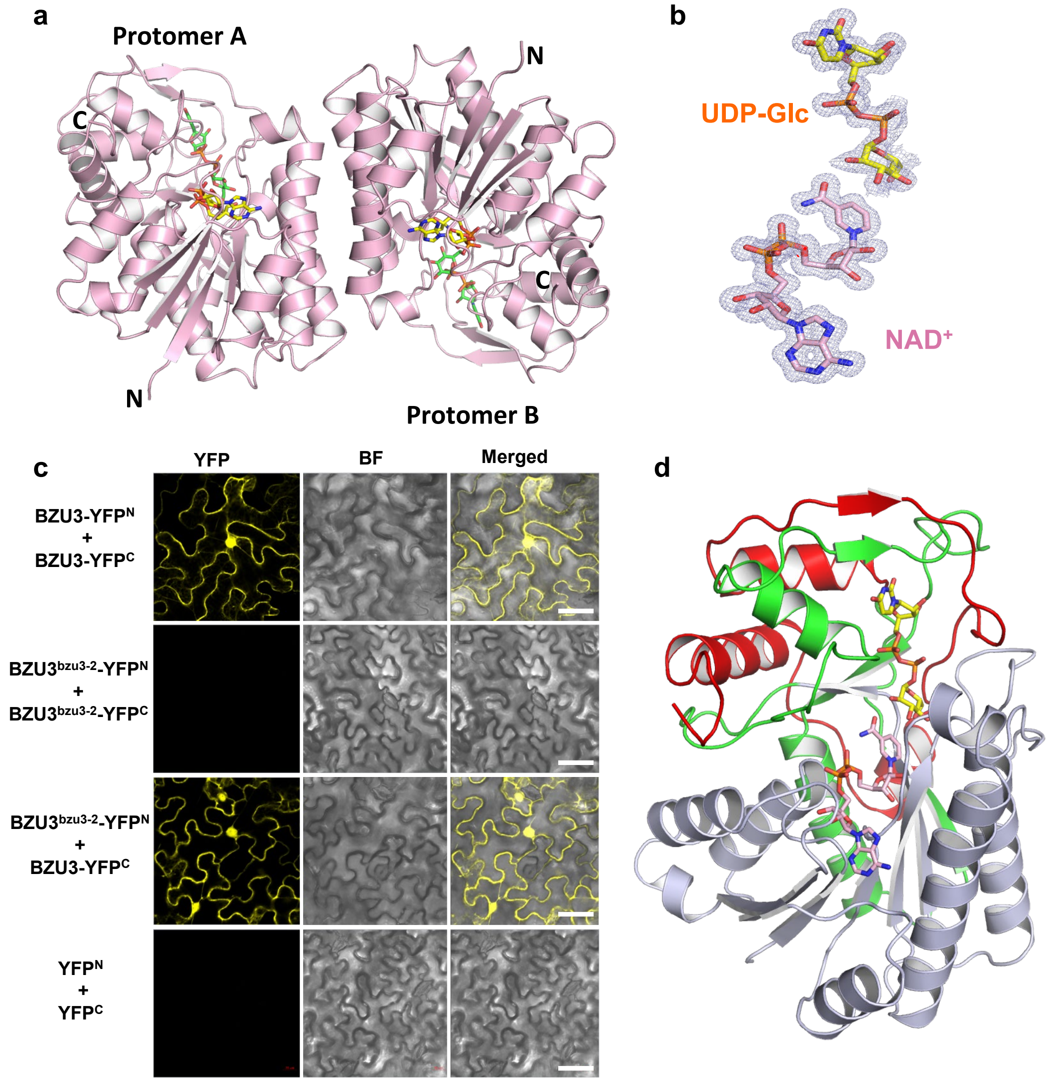

Fig. S16 Homo-dimerization structure of BZU3, and verification by BiFC *in vivo*.

a Dimeric structure of BZU3 in complex with NAD^+/UDP-Glc. The positions of NAD^+ (yellow) and UDP-Glc (green) were shown as stick models.

b Electron density maps of NAD^+ and UDP-glucose.

c BiFC assays to study the interactions of BZU3 and $BZU3^{bzu3\text{-}2}$. YFP^N and YFP^C (YFP halves) were used as negative control. Scale bar, 50 μm.

d The frameshift after T278 in *bzu3-2* was expected to alter the protein structure and the affected region was indicated in red. This change was predicted to impair substrate binding and catalytic activity of BZU3.

Supplementary Figure S17

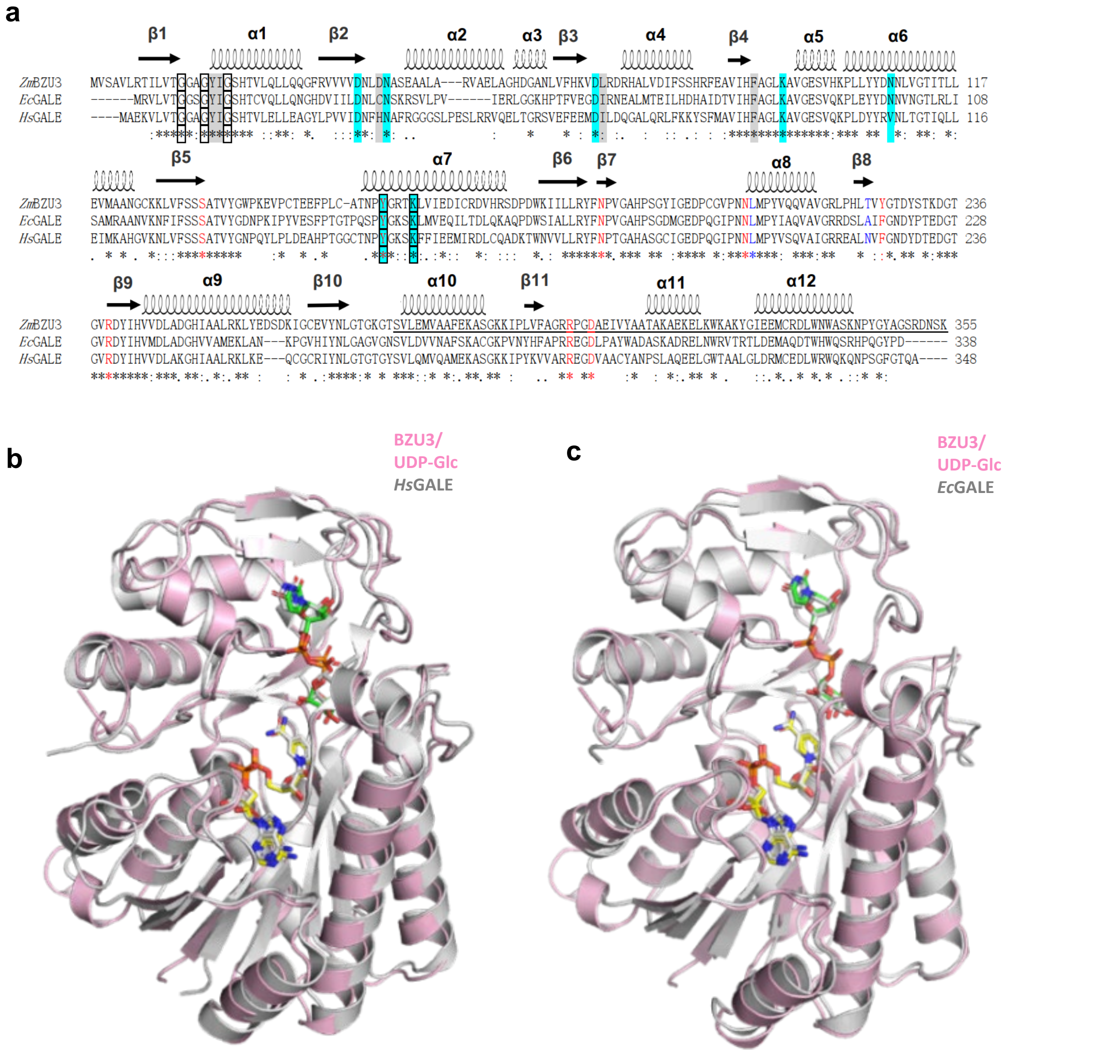

Fig. S17 Conservation of sequence and structure of BZU3.

a Structure-based sequence alignment for corn BZU3, bacterial *Ec*GALE, and human *Hs*GALE. Coils and arrows respectively define the α-helix and β-sheet elements in the BZU3 structure. The signature motifs “GXXGXXG” and “YXXXK” for NAD^+ binding are indicated in the boxes. The backbone- and sidechain-interacting residues for NAD^+ binding are highlighted in grey and cyan, respectively. The backbone- and sidechain-interacting residues for UDP-Glc binding are indicated in blue and red, respectively.

b, c Structural superimposition of BZU3 (UDP-Glc) with *Hs*GALE (E, PDB ID 1hzj) and *Ec*GALE (D, PDB ID 1xel), with r.m.s.d of 0.73 Å /331 C_{α} and 1.00 Å /331 C_{α}, respectively.

Supplementary Figure S18

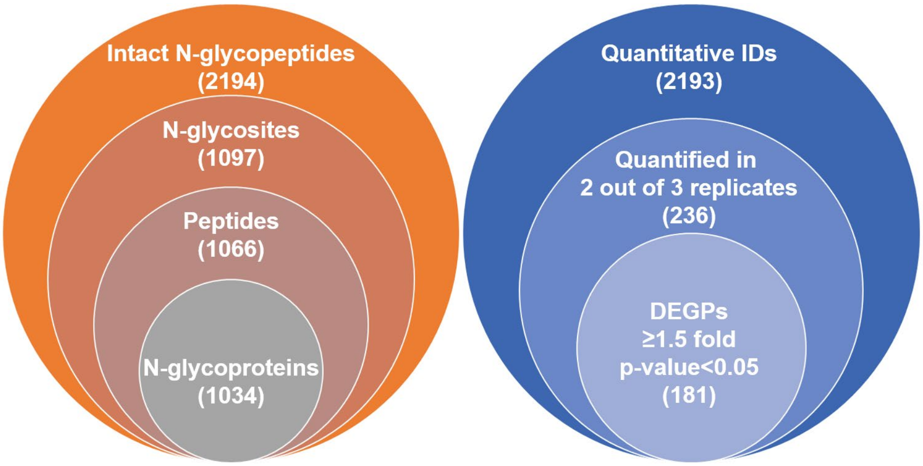

Fig. S18 Summary of the glycomics analysis of N-glycan types on the cell surface between the groups of *bzu3-2* and Zheng58.

Number of intact N-glycopeptides, N-glycosites, peptides and N-glycoproteins identified (left plot). Quantification and differential expressed results of N-glycopeptides (DEGPs) (right plot).

Supplementary Figure S19

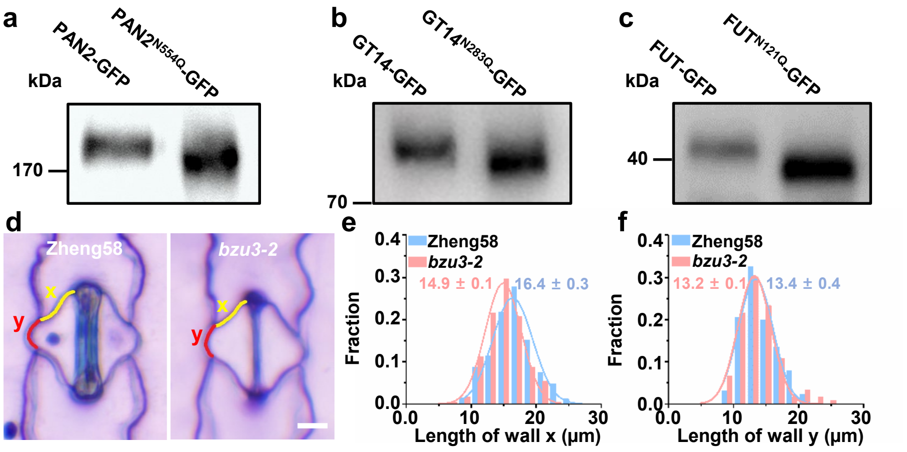

Fig. S19 N-glycosylation sites of several proteins were verified by gel shift assays and the abnormal subsidiary cell shape of *bzu3* mutants.

a-c Western blot verification of PAN2-GFP and PAN2^{N554Q}-GFP **(a)**, GT14-GFP and GT14^{N283Q}-GFP **(b)**, FUT-GFP and FUT^{N121Q}-GFP **(c)**. *35S:PAN2-GFP* and *35S:PAN2^{N554Q}-GFP*, *35S:GT14-GFP* and *35S:GT14^{N283Q}-GFP*, *35S:FUT-GFP* and *35S:FUT^{N121Q}-GFP* was expressed transiently in tobacco leaves respectively. After 48 h, the expression total proteins were extracted and analyzed through Western blot. PAN2: Zm00001d007862, GT14: Zm00001d008513, FUT: Zm00001d014505.

d Representative stomatal images of Zheng58 and *bzu3-2*. Wall x is the inter-stomatal cell shared with subsidiary cells (marked with yellow lines). Wall y is the flanking non-stomatal row cells shared with subsidiary cells (marked with red lines). The leaf epidermises were stained using toluidine blue. Scale bar, 10 μm.

e, f Statistical analysis of the wall x and y's lengths shown in **(d)**. The data was obtained by counting guard cells from six seedlings of each genotype (Zheng58: *n* = 126, *bzu3-2*: *n* = 120). Gaussian distributions were used to fit these histograms.

Source data are provided as a Source Data file.

Supplementary Figure S20

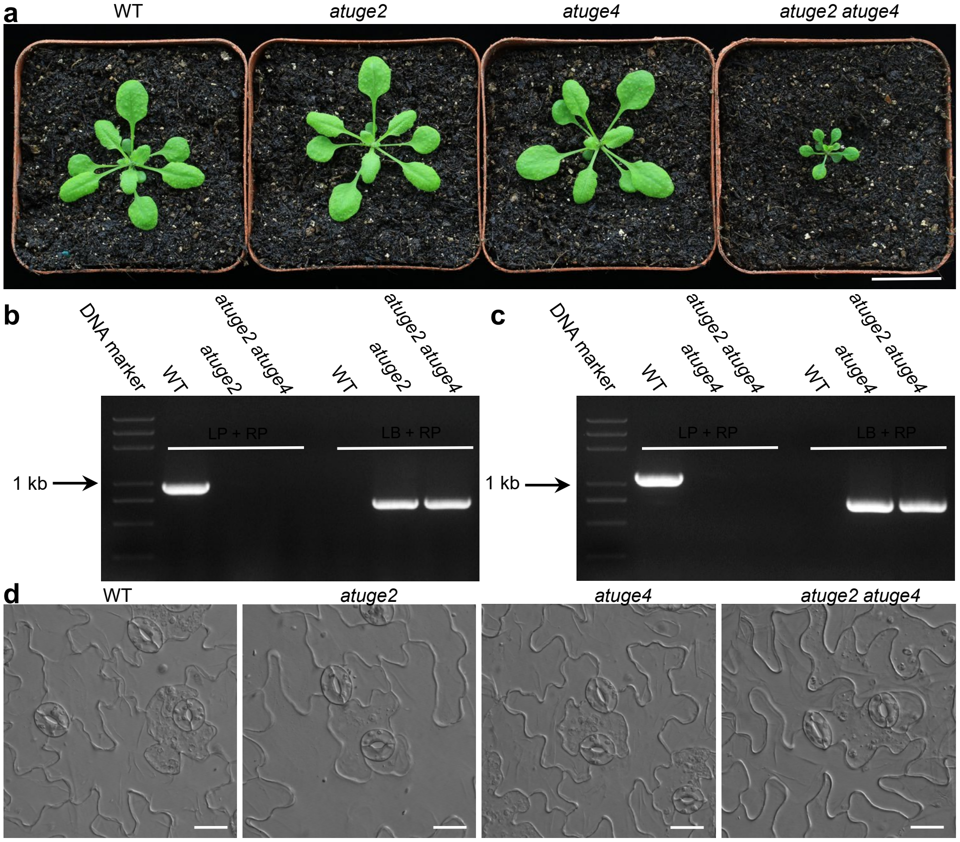

Fig. S20 Stomatal observations and molecular identification of WT, *atuge2*, *atuge4*, and *atuge2 atuge4*.

a Phenotypic analysis of 21 DAG WT, *atuge2*, *atuge4*, and *atuge2 atuge4*. Experiments were repeated three times independently with similar results. dag, days after germination. Scale bar, 2 cm.

b, c Genotyping of WT, *atuge2*, *atuge4*, and *atuge2 atuge4* plants. Genomic DNAs collected from WT, *atuge2*, *atuge4*, and *atuge2 atuge4* plants were used for PCR with specific primers. Primers (LP + RP) detected the wild type *AtUGE2* or *AtUGE4* respectively, while primers (LB + RP) detected the T-DNA insertion. The PCR products were run on a 1% agarose gel.

d Differential interference contrast (DIC) observations of the epidermis in WT, *atuge2*, *atuge4*, and *atuge2 atuge4* (rosette leaf, 21 DAG). Experiments were repeated three times independently with similar results. DAG, days after germination. Scale bars, 20 μm.

Source data are provided as a Source Data file.

Supplementary Figure S21

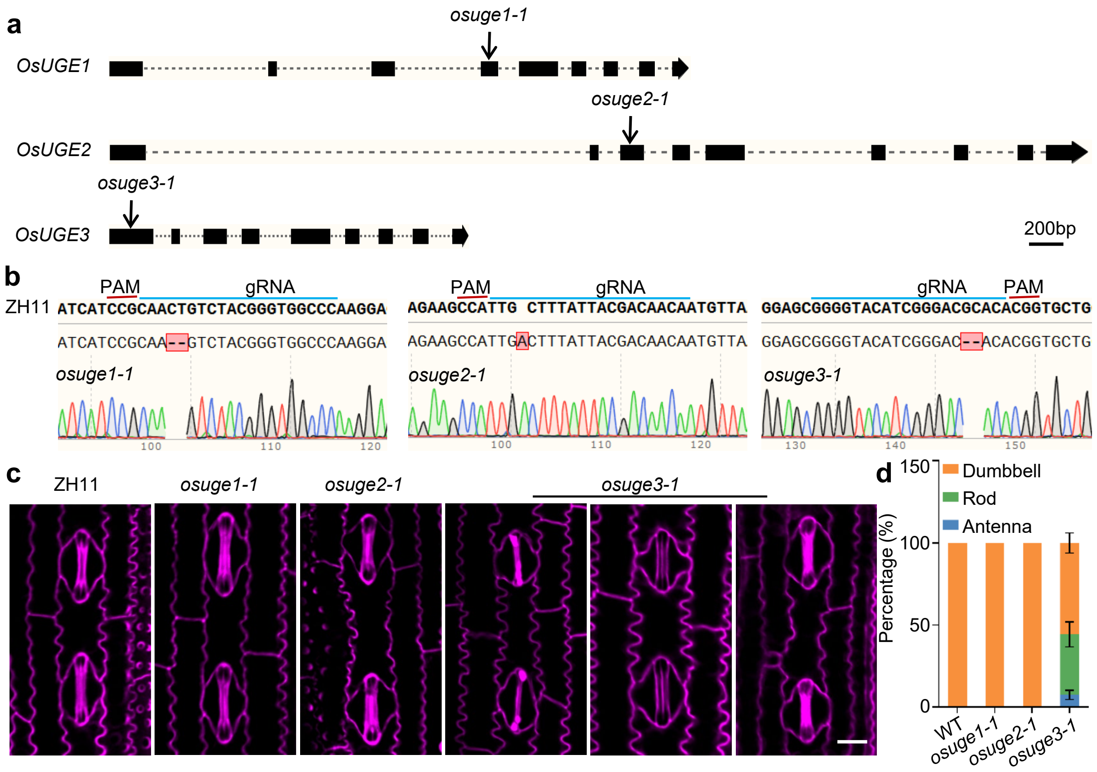

Fig. S21 Phenotypic analysis of CRISPR-induced mutations in *osuge1*, *osuge2*, and *osuge3*.

a Schematic representations of the genomic structures of *OsUGE1*, *OsUGE2*, and *OsUGE3*. The black boxes represent exons. CRISPR-induced mutants (*osuge1-1*, *osuge2-1*, and *osuge3-1*) are indicated on the gene modelsof *OsUGE1*, *OsUGE2*, and *OsUGE3*.

b Genotyping chromatograms of CRISPR-induced mutations of *osuge1-1*, *osuge2-1*, and *osuge3-1*. The target sequences and mutagenesis sequences are indicated for *OsUGE1*, *OsUGE2*, and *OsUGE3*. gRNA, guide RNA. PAM, protospacer adjacent motif.

c Stomatal phenotype of Zhonghua11 (ZH11) and CRISPR-Cas9-produced mutants (*osuge1-1*, *osuge2-1*, and *osuge3-1*). The images were obtained from an approximately 2-3 cm section taken from the second leaf of 10-12 dag plants. Propidium iodide (PI, purple) was used to stained cell walls. dag, days after germination. Scale bar, 10 μm.

d Proportional phenotypic scoring analysis of stomatal types in WT, *osuge1-1*, *osuge2-1*, and *osuge3-1.* The data was obtained by counting guard cells from five seedlings of each genotype (n = 640, 745, 648, and 766 respectively). Values represent means ± SD. SD, standard deviation. Source data are provided as a Source Data file.

Table S1. Segregation ratios of wild type plants and *bzu3-1* mutant plants in F_2. (*bzu3-1*/+♂ × ♀B73)

	Normal (N_1)	Abnormal (N_2)	$N_1 : N_2$
Real value	2421	779	3.11:1
Theoretical value	2400	800	3:1

$\chi^2 = 0.734 < \chi^2_{0.05} = 3.84$

Table S2. Segregation ratios of wild type plants and *bzu3-2* mutant plants in F_2. (*bzu3-2*/+♂ × ♀B73)

	**Normal (N_1)**	**Abnormal (N_2)**	**$N_1 : N_2$**
Real value	1008	340	2.96:1
Theoretical value	1011	337	3:1

$\chi^2 = 0.035 < \chi^2_{0.05} = 3.84$

Table S3. Segregation ratios of *bzu3-1* and *bzu3-2* mutant plants in crossed population F_1. (*bzu3-1/+*♂ × ♀*bzu3-2 /+*)

	Normal (N_1)	Abnormal (N_2)	$N_1 : N_2$
Real value	492	148	3.32:1
Theoretical value	480	160	3:1

$\chi^2 = 1.2 < \chi^2_{0.05} = 3.84$

Table S4. Kinetic parameters of BZU3 catalyzing conversions of UDP-Gal, UDP-Glc, UDP-GalNAc, and UDP-GlcNAc.

Substrate	K_m (mM)	V_{max} (mM·min^{-1})	K_{cat} (s^{-1})	K_{cat}/K_m (s^{-1}·mM^{-1})
UDP-Gal	1.45	1.41	942.0	650.3
UDP-Glc	2.67	0.69	460.6	172.4
UDP-GalNAc	0.50	0.36	238.8	474.7
UDP-GlcNAc	0.40	0.10	68.1	168.6

Table S5. Data collection and refinement statistics for BZU3 structures.

	NAD^+-bound BZU3<br><br>PDB ID: 7XPP	NAD^+/UDP-Glc-bound BZU3<br>PDB ID: 7XPO	NAD^+/UDP-GlcNAc-bound BZU<br>PDB ID: 7XPQ
**Data collection**			
Space group	C2	$P2_1$	$P2_1$
Cell dimensions			
a, *b*, *c* (Å)	163.04 88.149 82.431	60.933 84.657 73.514	65.873 84.945 73.436
α, β, γ (°)	90 117.241 90	90 109.321 90	90 110.695 90
Resolution (Å)	28.85 - 2.6 (2.693 - 2.6)*	27.22 - 1.25 (1.295 - 1.25)*	19.95 - 2.15 (2.227 - 2.15)*
R_{merge}	0.07544 (0.8546)	0.05982 (1.173)	0.2699 (3.254)
$I / \sigma I$	13.36 (1.49)	15.81 (1.40)	6.42 (0.59)
Completeness (%)	98.15 (98.07)	98.93 (98.02)	96.68 (92.35)
Multiplicity	6.8 (6.8)	6.6 (6.5)	7.0 (7.1)
**Refinement**			
Resolution (Å)	28.85 - 2.6	27.22 - 1.25	19.95 - 2.15
No. reflections	31493 (3101)	191937 (18981)	39884 (3777)
R_{work} / R_{free}	0.2484 / 0.2727	0.1547 / 0.1802	0.2043 / 0.2512
No. atoms			
Protein	5206	5309	5198
Ligand/ion	88	176	158
Water	0	770	405
B-factors			
Protein	106.90	17.29	35.06
Ligand/ion	93.41	20.02	36.78
Water	77.21	30.08	40.50
R.m.s. deviations			
Bond lengths (Å)	0.002	0.012	0.008
Bond angles (°)	0.53	1.23	1.13

*Values in parentheses are for the highest-resolution shell.

Table S6. Mutagenesis of BZU3 for enzyme activity tests.

	Amino acid	Mutagenesis site	Mutagenesis amino acid
S133A	Ser	133	Ala
Y157F	Tyr	157	Phe

Table S7. List of primers used in this study.

Experiment	Primer name	Primer sequence (5'-3')
RT-qPCR	*ubi2*-F	TGGTTGTGGCTTCGTTGGTT
	ubi2-R	GCTGCAGAAGAGTTTTGGGTACA
	BZU3-qRT-1F	CTCTCTCTCACACACACACATC
	BZU3-qRT-1R	AGGTCAACCTTGTGGAAGAC
	BZU3-qRT-2F	TCTACGAAGACTCCGACAAAAT
	BZU3-qRT-2R	GATTTTCTTCCCAGAAGCCTTC
	BZU3-qRT-3F	CTCGTCTTCCACAAGGTTGA
	BZU3-qRT-3R	AAGTGAATGACAGCCTCGAA
	BZU3-qRT-4F	TTTACTACGACAACAACCTGGT
	BZU3-qRT-4R	ATAGACAGTTGCAGATGACGAG
	BZU3-qRT-5F	GAAGGCTTCTGGGAAGAAAATC
	BZU3-qRT-5R	CTTCCATTTGAGCTCCTTCTCT
CRISPR/Cas9 constructs	Cas9-*BZU3*-T1-F	AATAATGGTCTCAGGCGGCGCAGAGCGGGAATTCTT
	Cas9-*BZU3*-T1-F0	GGCGCAGAGCGGGAATTCTTGTTTTAGAGCTAGAAATAGC
	Cas9-*BZU3*-T2-R0	TATGGGCGGACAAAGGTTCCGCTTCTTGGTGCC
	Cas9-*BZU3*-T2-F	ATTATTGGTCTCTAAACTATGGGCGGACAAAGGTTC
BZU3pro:BZU3-YFP	*BZU3pro:BZU3-YFP*-1F	gcaggtcgactctagaagcttTATGCGAACGGTCTGACTGG
	BZU3pro:BZU3-YFP-1R	cggacaccatCGTCGGTAGGCCCTCGGA
	BZU3pro:BZU3-YFP-2F	cctaccgacgATGGTGTCCGCCGTGCTC
	BZU3pro:BZU3-YFP-2R	tatttaaatggatccggcgcgcTTTGCTGTTGTCGCGTGACC
BZU2pro:BZU3-YFP	*BZU2pro:BZU3-YFP*-1F	gcaggtcgactctagaagcttATAGGAAAAAGAATCACTAAATTCAGAGT
	BZU2pro:BZU3-YFP-1R	cggacaccatAATGTGATCGATCGACGACTACTG
	BZU2pro:BZU3-YFP-2F	cgatcacattATGGTGTCCGCCGTGCTC
	BZU2pro:BZU3-YFP-2R	tatttaaatggatccggcgcgcTTTGCTGTTGTCGCGTGACC
FAMApro:YFP-BZU3	*FAMApro:YFP-BZU3*-1F	cggatccatttaaatactagtATGGTTTTGCAACCGACGAC
	FAMApro:YFP-BZU3-1R	atggtggatcccatGATCAGGCGGGTGGAGCA
	FAMApro:YFP-BZU3-2F	ctgatcATGGGATCCACCATGGTGAGC
	FAMApro:YFP-BZU3-2R	cggacaccatTCTAGAGGATCCGTTCAAGTCTTCT
	FAMApro:YFP-BZU3-3F	atcctctagaATGGTGTCCGCCGTGCTC
	FAMApro:YFP-BZU3-3R	cgatcggggaaattcgagctcTCATTTGCTGTTGTCGCGTG
Prokaryotic protein expression	His-BZU3-F	cagcaaatgggtcgcggatccATGGTGTCCGCCGTGCTC
	His-BZU3-R	ctcgagtgcggccgcaagcttTCATTTGCTGTTGTCGCGTG
Site-directed mutation	S133A-F	GCTGGTGTTCTCGTCAGCTGCAACTGT
	S133A-R	CTGACGAGAACACCAGCTTCTTGCAGC
	Y157F-F	TGCGCCACCAATCCCTTTGGGCGGACAAA
	Y157F-R	AAGGGATTGGTGGCGCAGAGCGGGAATT
Arabidopsis mutant identification primers	*atuge2*-LP	CTATCCCATTCTTAGCCCTGC
	atuge2-RP	ACATCGTTCTGACTCGGAATG
	atuge4-LP	AGGACATTTGCCGTGATGTAC
	atuge4-RP	CTATGCCAAAACAGGTCCATG
Rice CRISPR/cas9 mutants identification primers	*osuge1*-F	GCGGCATCGCAACAGATTTA
	osuge1-R	CCTCACCAATGTACCCGCTT
	osuge2-F	TGTGCAGGGTGAGTTCTTCG
	osuge2-R	CAGAGGCATGCCCATCATCA
	osuge3-F	TGCTCTTGAGATCCCCCTGA
	osuge3-R	CGTCCGCTGCGAACTACTAA
BiFC assay	BZU3/BZU3$^{bzu3\text{-}2}$-YFP^{C}-F	attacaggtacccggggatccATGGTGTCCGCCGTGCTC
	BZU3-YFP^{C}-R	cacgctgccaccgccgtcgacTTTGCTGTTGTCGCGTGACC
	BZU3$^{bzu3\text{-}2}$-YFP^{C}-R	cacgctgccaccgccgtcgacATTAATTATGGTAGCATAGGCTATAAGG
	YFP^{N}-BZU3/BZU3$^{bzu3\text{-}2}$-F	atcgaggacgccggcggatccATGGTGTCCGCCGTGCTC
	YFP^{N}-BZU3-R	acgaaagctctgcaggtcgacTCATTTGCTGTTGTCGCGTG
	YFP^{N}-BZU3$^{bzu3\text{-}2}$-R	acgaaagctctgcaggtcgacCTAATTAATTATGGTAGCATAGGCTATAAGG
35S:PAN2-GFP	*35S:PAN2-GFP*-F	ggacagggtacccggggatccATGGGGCTTCGCGCGGGT
	35S:PAN2-GFP-R	agctcctcctcctctctagaGATCGACGAAAGATCCTCGTACA
PAN2 mutation	PAN2^{N554Q}-F	CTCAACAAACCAATTCTCTGGCCCACTACCTGCTG
	PAN2^{N554Q}-R	GGCCAGAGAATTGGTTTGTTGAGATGTCGAGAACTC
35S:GT14-GFP	*35S:GT14-GFP*-F	attacaggtacccggggatccATGGGCGCCGCCGACAAG
	35S:GT14-GFP-R	catggtggctcccatggatccTTGCTTGCAAGAGTTGTTGCC
GT14 mutation	GT14^{N283Q}-F	CTTCCGCcaaTTCACAGTGAACAACTACTTGCGG
	GT14^{N283Q}-R	CTGTGAAttgGCGGAAGTCCGAGTTGCATGCA
35S:FUT-GFP	*35S:FUT-GFP*-F	attacaggtacccggggatccATGTATCGCCGAAACCCG
	35S:FUT-GFP-R	catggtggctcccatggatccTTGTGGGCGCGTGTCGAA
FUT mutation	FUT^{N121Q}-F	GCGGGAAcaaTTCAGCTTCGACACGCGCCCAC
	FUT^{N121Q}-R	AGCTGAAttgTTCCCGCTGTGGCAATTCAGCT
