# Supplementary material for: A maize epimerase modulates cell wall synthesis and glycosylation during stomatal morphogenesis
Source: Nat Commun. 2023 Jul 20;14:4384. doi: 10.1038/s41467-023-40013-6 (PMC10359280; doi:10.1038/s41467-023-40013-6)
Supplement: Supplementary file 3 — Description of Additional Supplementary Files [file 41467_2023_40013_MOESM3_ESM.pdf]

Description of Additional Supplementary Files

Supplementary Data S1. Differentially expressed intact N-glycopeptides (DEGPs).
